# Modeling allelic diversity of multiparent mapping populations affects detection of quantitative trait loci

**DOI:** 10.1093/g3journal/jkac011

**Published:** 2022-01-17

**Authors:** Sarah G Odell, Asher I Hudson, Sébastien Praud, Pierre Dubreuil, Marie-Hélène Tixier, Jeffrey Ross-Ibarra, Daniel E Runcie

**Affiliations:** 1 Department of Plant Sciences, University of California, Davis, CA 95616, USA; 2 Department of Evolution and Ecology, University of California, Davis, CA 95616, USA; 3 Center for Population Biology, University of California, Davis, CA 95616, USA; 4 Limagrain, Centre de Recherche de Chappes, Chappes 63720, France; 5 Genome Center, University of California, Davis, CA 95616, USA

**Keywords:** MAGIC, QTL, linkage mapping, association mapping, MPP, multiparental populations, multiparent advanced generation intercross (MAGIC)

## Abstract

The search for quantitative trait loci that explain complex traits such as yield and drought tolerance has been ongoing in all crops. Methods such as biparental quantitative trait loci mapping and genome-wide association studies each have their own advantages and limitations. Multiparent advanced generation intercross populations contain more recombination events and genetic diversity than biparental mapping populations and are better able to estimate effect sizes of rare alleles than association mapping populations. Here, we discuss the results of using a multiparent advanced generation intercross population of doubled haploid maize lines created from 16 diverse founders to perform quantitative trait loci mapping. We compare 3 models that assume bi-allelic, founder, and ancestral haplotype allelic states for quantitative trait loci. The 3 methods have differing power to detect quantitative trait loci for a variety of agronomic traits. Although the founder approach finds the most quantitative trait loci, all methods are able to find unique quantitative trait loci, suggesting that each model has advantages for traits with different genetic architectures. A closer look at a well-characterized flowering time quantitative trait loci, qDTA8, which contains *vgt1*, highlights the strengths and weaknesses of each method and suggests a potential epistatic interaction. Overall, our results reinforce the importance of considering different approaches to analyzing genotypic datasets, and shows the limitations of binary SNP data for identifying multiallelic quantitative trait loci.

## Introduction

The study of quantitative genetics requires the ability to link differences in phenotype to genotypic variation. Natural and artificial selection act on phenotypes, but only heritable phenotypic variation will result in changes in population means. Maize presents an excellent model organism to study quantitative genetics due to the combination of extensive genetic and phenotypic resources and the ability to create mapping populations. In addition, maize is one of the most widely produced crops in the world and is a major source of calories for millions of people. Decades of research into maize genetics have resulted in the identification of many quantitative trait loci (QTL) that explain variation in phenotypes such as yield, flowering time (FT), and plant height (PH) (Beavis et al. 1991; Wang et al. 2006; Buckler et al. 2009; Steinhoff et al. 2012; Wallace et al. 2014). Such traits are extremely agronomically important, and are also crucial in terms of fitness and local adaptation. Researchers have discovered large-effect QTL for a number of agronomic traits in maize through the use of different types of mapping populations (Huang et al. 2015).

The choice of mapping population comes with associated advantages and limitations. In particular, different types of populations tend to vary in 2 main characteristics: (1) their ability to capture genetic diversity and (2) their power to detect QTL of small effect. Multiparent advanced generation intercross (MAGIC) populations have been used in breeding to increase the genetic diversity included in a mapping population compared to biparental populations in multiple model organisms. In plants, MAGIC populations have been created for maize (Dell’Acqua et al. 2015; Anderson II et al. 2018; Jiménez-Galindo et al. 2019; Liu et al. 2020), wheat (Huang et al. 2012), tomato (Pascual et al. 2015), and Arabidopsis (Kover et al. 2009; Huang et al. 2011), among others. For animal models, populations such as the Collaborative Cross in mice (Churchill et al. 2004; Aylor et al. 2011) and the *Drosophila* Synthetic Population Resource (King et al. 2012) have likewise been created. Compared to genome-wide association panels, MAGIC populations have more power to detect low frequency alleles and can better compare allelic effects between founders because the crossing scheme increases the frequency of all parental alleles to be approximately equal. Simulations of an 8-parent MAGIC population showed that sample sizes of 300 could detect QTL accounting for 12% of variance with a power of 82% averaged across minor allele frequencies (Dell’Acqua et al. 2015).

In this study, we used a MAGIC population of 344 doubled haploid (DH) lines derived from 16 inbred maize parents developed by the company Biogemma to understand how genetic models can impact the identification of QTL. Compared to previous populations in maize, this MAGIC population has a greater number of founders, and should have comparable power to larger nested mapping populations (Yu et al. 2008; Dell’Acqua et al. 2015). In addition, using DHs instead of recombinant inbred lines (RILs) removes any residual heterozygosity, ensuring that replicates are genetically identical. For these reasons, the Biogemma MAGIC population that we present here has great potential to reveal new insights into the genetic control of quantitative traits in maize.

In addition to the choice of mapping population, the choice of how to model allelic variation can impact the power of a study to detect and analyze QTL. A bi-allelic model for QTL, often used in genome-wide association studies (GWAS), assumes that the underlying causal variants for QTL are explained by 2 alleles, usually SNPs, that are segregating in the population. With this bi-allelic model, hereafter referred to as *GWAS_SNP_*, markers represent a small region of the chromosome that is in tight linkage disequilibrium with the genotyped SNP.

An alternative model for the allelic state of QTL can be used in multiparent populations, where we assume that each founder contributes its own allele. In this model, comparable to interval mapping, rather than looking at individual SNPs, an allele becomes the founder identity of that region, or which parent of the population that segment of chromosome was derived from. As a result, QTL are multiallelic, with the number of alleles equal to the number of founders used in the making of the population. We will refer to this founder model hereafter as *QTL_F_*.

The 2 allelic models described above make the assumption that for each genotyped SNP, there are either 2 distinct alleles in the population (*GWAS_SNP_*) or as many alleles as there are founders (*QTL_F_*). The latter assumption, although very possible for biparental mapping populations, becomes increasingly unlikely as the number of founders increases. This is because the founders used in the making of a population are related to one another with varying degrees of distance, and therefore, most likely share ancestral haplotypes through identity-by-descent (IBD). A third allelic model takes into account shared ancestral haplotypes between founders. This model, hereafter referred to as *QTL_H_*, allows the number of alleles at each site to vary anywhere from 2 to the total number of founders (here 16), based off of the number of ancestral haplotypes at that site. This has the potential to increase statistical power compared to the *QTL_F_* model by reducing the number of parameters that must be estimated by the model.

Here, we present a maize MAGIC population derived from 16 parents and discuss the performance of 3 different models for representing allelic states: bi-allelic, founder, and ancestral haplotype allelic models for detecting QTL. Using *vgt1*, a well-characterized FT QTL with a strong candidate causal variant that is variable in the population, we demonstrate differences between the 3 methods and explore potential interactions between *vgt1* and other genetic variation in the population.

## Materials and methods

### Mapping population

**Fig. 1.**
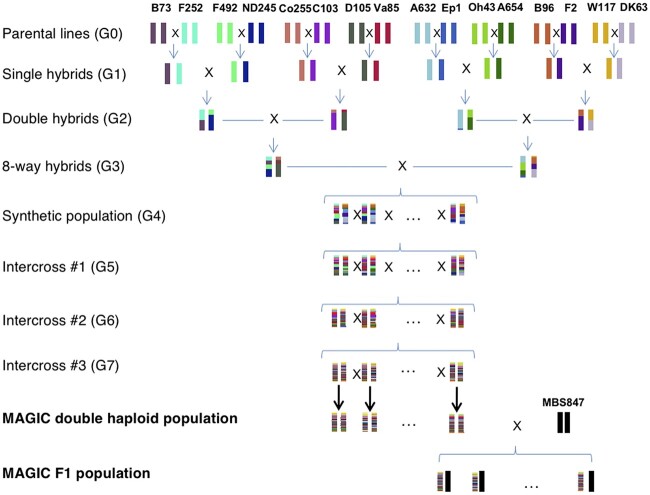
The crossing scheme of the MAGIC population; 16 inbred maize lines were crossed in a funnel crossing scheme, and then the 8-way hybrids were outcrossed for 3 generations. The outcrossed lines were then made into DHs, which where were crossed to an inbred tester, MBS847 to make MAGIC F1s.

The MAGIC population was derived from 16 inbred maize parents representing the diversity of temperate maize (Supplementary File 1). The parents were chosen to be a mix of members of Flint and Dent heterotic groups, which are historically and genetically diverged groups that, when crossed, produce F1 plants that display hybrid vigor. In addition, the founders were chosen to represent a phenotypically diverse set of FTs for temperate maize. The 16 founder lines were crossed in a funnel crossing scheme, and then the resulting synthetic population was intercrossed for 3 generations with around 1,600 individuals per cycle (Fig. 1). The founder pairs crossed in the initial F1 stage were chosen to cross early and late flowering lines to one another, with the intention of maintaining genetic and phenotypic diversity in FT, and this process was continued throughout (Supplementary File 1). Finally, 800 lines were selected from the synthetic population to create DHs, resulting in 550 MAGIC DH lines at the end of the process. The MAGIC DH lines were crossed to a tester MBS847 to produce 344 hybrids (Fig. 1).

### Genotype data

The 16 founder lines and the MAGIC DH lines were all genotyped with the Affymetrix 600K Axiom SNP array (Unterseer et al. 2014), resulting in genotype data for 517,769 SNPs. A total of 503,902 SNPs were used from the 600K after filtering out invariant sites and sites that were not located on autosomal chromosomes according to the B73 AGPv4 reference genome (Jiao et al. 2017). Linkage disequilibrium was calculated as *R*^2^ of pairwise SNPs using the software PLINK and the 600K SNP data (Supplementary Fig. 1) (Purcell et al. 2007).

### Phenotype data

The MAGIC F1 plants were phenotyped in 5 different field locations in 4 different years, resulting in 6 distinct environment-years (Hudson et al. in prep). The environments included Blois, France (2014 and 2017), Graneros, Chile (2015), Nerac, France (2016), St. Paul, France (2017), and Szeged, Hungary (2017). The environments represent a range of latitudes and water stress, from vegetative and flowering water deficit (Nerac, 2016) to optimum well-watered conditions (Graneros, 2015). In each environment, we grew a minimum of 292 and a maximum of 309 of the DH lines. Each genotype was grown with 2 replicates in each environment. In all environments, 6 traits were measured: grain yield, PH, female flowering date (DTS), male flowering date [days to anthesis (DTA)], thousand kernel weight (TKW), and harvest grain moisture (HGM). By subtracting male flowering date from female flowering date, we also obtained anthesis–silking interval. For each of the lines, we calculated best linear unbiased predictor (BLUP) scores for all 7 phenotypes, combining measurements from all environments to get estimates of the genetic contribution to the phenotype for each MAGIC line (Aulchenko et al. 2007).

### Calculation and validation of founder probabilities

We used the package R/qtl2 (Broman et al. 2019) to determine founder probabilities of the MAGIC DH lines using the 600K genotype data and the cross type “riself16”. We then filtered down the founder probabilities from all 503,902 sites to represent founder recombination blocks for interval mapping. Markers for the *QTL_F_* approach were filtered based on linkage disequilibrium using an iterative approach where a marker was dropped if the *R*^2^ value of the founder probabilities between it and the previous marker was greater than 0.95. After filtering, a total of 4,578 sites were kept to represent founder recombination blocks in the MAGIC DH lines.

**Fig. 2.**
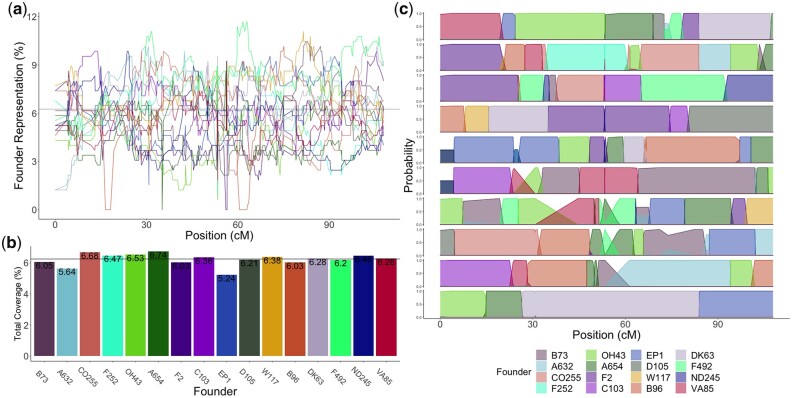
Founder representation of the MAGIC population. a) Coverage of each founder across the population on chromosome 10 as a percentage. Black line represents expected number of lines per founder with equal distribution (6.25%). Each of the 16 founders are indicated by a color. b) Total coverage of each founder across the population as a percentage. Black line shows expectation of equal distribution (6.25%). c) Founder probabilities for 10 individual MAGIC DH lines on chromosome 10 in genetic distance.

Due to the fact that the actual crossing scheme and the cross type input into R/qtl2 differed (DH lines rather than RILs), we wanted to assess the accuracy of the founder probabilities. This was done by simulating lines using the actual crossing scheme and assessing the performance of the calc_genoprobs function of R/qtl2 in correctly identifying the founder genotype (Fig. 2c). We developed an R package (R Core Team 2017), *magicsim* (https://github.com/sarahodell/magicsim) to simulate the lines using the maize consensus genetic map from Ogut et al. (2015) to generate approximate recombination rates across the chromosome. We simulated 100 MAGIC populations constituting 325 lines and assessed founder assignment accuracy as the average percentage of SNPs where the predicted founder was the same as the actual founder.

### Test for equal representation of alleles

The number of lines that had a probability of a particular founder greater than 0.8 were used as an approximation of the number of lines that had that founder at a site. This observed count was compared to a null expectation of 1/16 for equal distribution across lines (approximately 21 lines per founder) (Fig. 2, a and b). We performed a $\chi^{2}$ test for each site to determine if founder counts significantly deviated from null expectation. We obtained a 5% significance threshold using the $\chi^{2}$ obtained from founder counts in 100 simulated populations. The $\chi^{2}$ tests from simulated lines were done using reconstructed genotype data pulled from the 600K genotype data of the 16 founders. We then used the same methods of calculating founder probabilities with R/qtl2 used with the actual population. Due to the fact that we used the inferred founder identities of the simulated lines, rather than the known founder identities, the null distribution of *P*-values generated from $\chi^{2}$ tests of the simulated populations incorporated uncertainty of founder assignment.

### Calculation of IBD and haplotype probabilities

The identification of regions of shared genetic sequence between founder pairs allows collapsing of founders into haplotypes. IBD was measured from the 600K SNP data of the founders using the software RefinedIBD with a sliding window of 10 cM and a minimum IBD segment length of 0.2 cM (Browning and Browning 2013). The resulting segments of pairwise IBD between each of the 16 founders were used to identify distinct haplotype blocks. We did this by moving along the chromosome, starting a new haplotype block when a segment of pairwise IBD between founders started or ended (Supplementary Fig. 2). Then, within blocks, we grouped all founders that were in IBD with one another into a haplotype. Within blocks, the founder probabilities for founders that shared a haplotype were summed to obtain haplotype probabilities.

In certain instances, the pairs of founders that were in IBD with one another in a particular haplotype block formed an incomplete graph, where not all founders were in IBD with all other founders (Supplementary Fig. 2). For example, from the results of RefinedIBD, founder A was in pairwise IBD with both founder B and founder C, but founder B and C were not in pairwise IBD.

For the sake of simplicity, we continued with the assumption that all founders in a haplotype were in IBD with one another (we called B and C as in IBD). However, it is important to note that cutoffs for IBD are inherently arbitrary, and haplotypes called here do still possess genetic differences between founders, with some founders more different than others.

Markers for the *QTL_H_* mapping approach were filtered for LD using an iterative approach similar to *QTL_F_*: for all haplotype blocks with the same number of distinct haplotypes, a marker was dropped if the correlation of haplotype probabilities between it and the previous marker was greater than 0.95. After filtering, a total of 11,105 sites were kept to represent haplotype blocks in the MAGIC DH lines.

### Association and QTL mapping

The R package GridLMM (Runcie and Crawford 2019) was used to run association mapping using the 3 different models of representing the QTL allelic state. The function GridLMM_ML was used with the “ML” option. The following 3 models were approximated by fitting each locus independently. The 3 methods differed in the *X* matrix used in the mixed linear model. The bi-allelic model (*GWAS_SNP_*) is:

(1) $$y=\mu +x_{Si}\beta_{Si}+Zu+\epsilon$$

where *y* is the response variable, *μ* is the global mean, *x_Si_* is an *n* × 1 genotype vector for SNP *i* with reference and alternate alleles represented as 0 and 1, respectively, *β_Si_* is the effect size of the alternate allele, *Z* is the design matrix, $u∼N(0,\sigma_{u}^{2}K)$ is the random effects of markers across the rest of the genome using the genomic relationship matrix, *K*, and *ϵ* is the error. The genomic relationship matrix, *K* was generated from the 600K SNP data using a leave-one-chromosome-out method. The same *K* matrix was used for all 3 models.

The founder model (*QTL_F_*) is:

(2) $$y=\mu +X_{Fi}\beta_{Fi}+Zu+\epsilon$$

where *X_Fi_* is a *n* × *f* – 1 matrix for marker *i* and *x_fni_* is the probability that at site *i*, individual *n* was derived from founder *f*, and *β_Fi_* is the effect size of each founder allele.

The ancestral haplotype model (*QTL_H_*) is:

(3) $$y=\mu +X_{Hi}\beta_{Hi}+Zu+\epsilon$$

where *X_Hi_* is an *n* × *h* – 1 matrix for marker *i* and *x_hni_* is the probability that at site *i*, individual *n* has ancestral haplotype *h* and *β_Hi_* is the effect size of each haplotype allele. Significance cutoffs for *P*-values were obtained using permutation testing, taking the 5% cutoff from 1,000 permutations where genotypes were randomized relative to phenotypes for each method. For the 2 largest QTL peaks, *qDTA3-2* and *qDTA8*, we tested if the peaks contained more than 1 QTL by including the most significant site as a covariate and testing to see if any other sites within the support interval remained significant.

### Model comparison

The results of the 3 models were compared using 2 main criteria: (1) presence or absence of identified QTL peaks and (2) the size of QTL support intervals. QTL support intervals were determined by identifying the most significant SNP for a QTL peak and demarcating the left and right bounds of the QTL as the left-most and right-most SNPs within a 100-Mb window centered on the highest SNP that have a –log10(*P*-value) that is 2 log10(*P*-values) below that of the highest SNP. The detection of QTL was compared across the 3 methods for each phenotype. A QTL was said to be identified across models if the QTL support interval for that QTL overlapped. The effect of the model used on the size of QTL support intervals was investigated using the QTL which were identified by all 3 methods at the 5% significance threshold (*n* = 26). The support interval size response variable was represented both in terms of physical distance (Mb) and genetic distance (cM).

### Estimation of effect sizes

We used the R package *lme4qtl* to calculate standard errors of effect sizes relative to the population mean (Ziyatdinov et al. 2018). For the *QTL_F_* model, effect sizes were dropped for individual founders at some sites if there were fewer than 5 MAGIC lines that had a probability greater than 0.8 for 1 of the founders. This same filtering was done with *QTL_H_* effect sizes for sites with low representation of particular haplotypes. This was to ensure that effect sizes for individual founders and haplotypes could be effectively estimated. We confirmed that effect sizes calculated by GridLMM and lme4qtl matched one another, with the correlations in effect sizes between the 2 methods greater than 0.99.

### Tests for epistasis

We ran a genome scan for epistatic interactions with *vgt1*. The probability of the MAGIC lines having the MITE insertion at *vgt1* was calculated by summing the founder probabilities for all founders that have the *MITE*^+^ allele at the site closest to the location of the MITE underlying *vgt1* in the B73 APGv4 assembly found on MaizeGDB (Portwood et al. 2019). Lines that had uncertain allelic states at the MITE [0.05 > Pr(*MITE*^+^) < 0.95] were dropped for the test. Applying a Bonferroni significance threshold adjusted for the number of tests, we tested for epistasis using the 600K genotype data.

We also performed QTL mapping with *QTL_F_* using only the *MITE*^+^ MAGIC lines. This was to see if there were any other loci whose effect was only observed in the presence of the MITE. A normal epistatic model could not be fit with founder alleles because there were not enough degrees of freedom to compare each founder. We used the model from Equation (2) using DTA BLUP scores and the 5% significance threshold for DTA.

### FT enrichment tests

We used a list of FT genes assembled by Wang et al. (2017) to test for enrichment of FT genes in (1) regions that had significantly uneven representation of the founders and (2) regions with high interchromosomal LD (*R*^2^ > 0.9). Of the 907 genes, we used 887 which were aligned to autosomal chromosomes in the B73 AGPv4 assembly (Jiao et al. 2017). To determine a null distribution, we randomly sampled 887 non FT genes and counted the number of genes that overlapped with these 2 sets of regions over 1,000 permutations. We compared this distribution to the actual number of FT genes that overlapped with the selected regions.

## Results

### MAGIC population

We developed a 16-parent MAGIC population using temperate inbred maize lines representative of the diversity of the Flint and Dent heterotic groups of North America and Europe (Fig. 1). We genotyped 344 MAGIC DH lines from the population with a 600K SNP genotyping array and measured 7 phenotypes across 6 environments from the MAGIC F1s. Using the phenotype data from the 6 environments, we calculated BLUP scores for each of the MAGIC lines. PCA analysis of the MAGIC lines and the 16 founders and tester suggested that the MAGIC lines maintained much of the genetic variation possessed by the founders, without overwhelming bias toward any particular founders (Supplementary Fig. 3). In addition, the minor allele frequencies of SNPs in the 16 founder compared to in the MAGIC lines suggested that lower frequency SNPs in the founders were either maintained or brought up in frequency in the MAGIC lines, which aids in the estimation of SNP effect sizes (Supplementary Fig. 4).

### Simulation and validation of founder probabilities

We partitioned the genomes of individual MAGIC lines into segments of ancestry from the 16 founders. This allowed us to determine the predicted contribution of each founder to the population (Fig. 2c). The founder probabilities determined using R/qtl2 were able to assign founders to the actual MAGIC DH lines with high confidence (> 0.80) for 96.7% of the 10 chromosomes of maize. The median size of recombination blocks was 4.32 Mb and the mean size was 15.76 Mb with a standard deviation of 29.52 Mb. The average number of crossover events per line was 123.7 with a standard deviation of 20.73. Our simulations suggest a very high (μ=99.8%,σ=0.011) assignment accuracy (see *Materials andmethods*). This reinforced our confidence in the founder probabilities obtained from the actual data.

### IBD and MAGIC haplotypes

In some cases, the model which inferred founder identity in the MAGIC lines had high uncertainty, with probabilities split approximately equally between 2 founders. We hypothesized that this uncertainty was due to the 2 founders having very similar genetic sequence at those regions, such that the model struggled to differentiate the 2. To assess the genetic similarity of the founders, we calculated pairwise IBD between all founders using the software RefinedIBD (Browning and Browning 2013). Areas of uncertainty in founder probabilities of the DH lines were associated with regions of IBD between 2 or more founder lines in that region of the chromosome (Supplementary Fig. 5).

The results showed that a total of 1.81 Gb (86%) and 1,367.5 cM (92.7%) of the genome were in IBD between at least 2 different founders. The average size of an IBD segment between 2 founders was 140 kb (0.51 cM) with a median of 122 kb (0.46 cM). Pairwise IBD segment sizes ranged from 8 kb (0.3 cM) to 673 kb (1.61 cM). For founder pairs that were found to be in IBD with one another, the total percentage of IBD between founders ranged from 0.0018% (F492 and VA85) to 4.39% (B73 and A632), with an average of 0.061%. There were no IBD segments found for 18 of 120 possible pairwise founder combinations. The amount of IBD segments between the 16 founders and the tester, MBS847, was mostly low (ranging from 0.14% of the genome for F2 to 3.7% for B73), with the notable exception of DK63, which was in IBD with MBS847 for 36.5% of the genome. The Neighbor-Joining Tree of the relatedness of the 16 founders and MBS847 recapitulated the IBD results (Supplementary Fig. 6). For a particular founder pair, B73 and A632, there were large segments where the lines shared haplotypes, and the tree placed them very close together. This is consistent with the known pedigree of the lines, where A632 was derived from B14, a line from the same heterotic group as B73 (Supplementary File 1) (Lorenz and Hoegemeyer 2013).

**Fig. 3.**
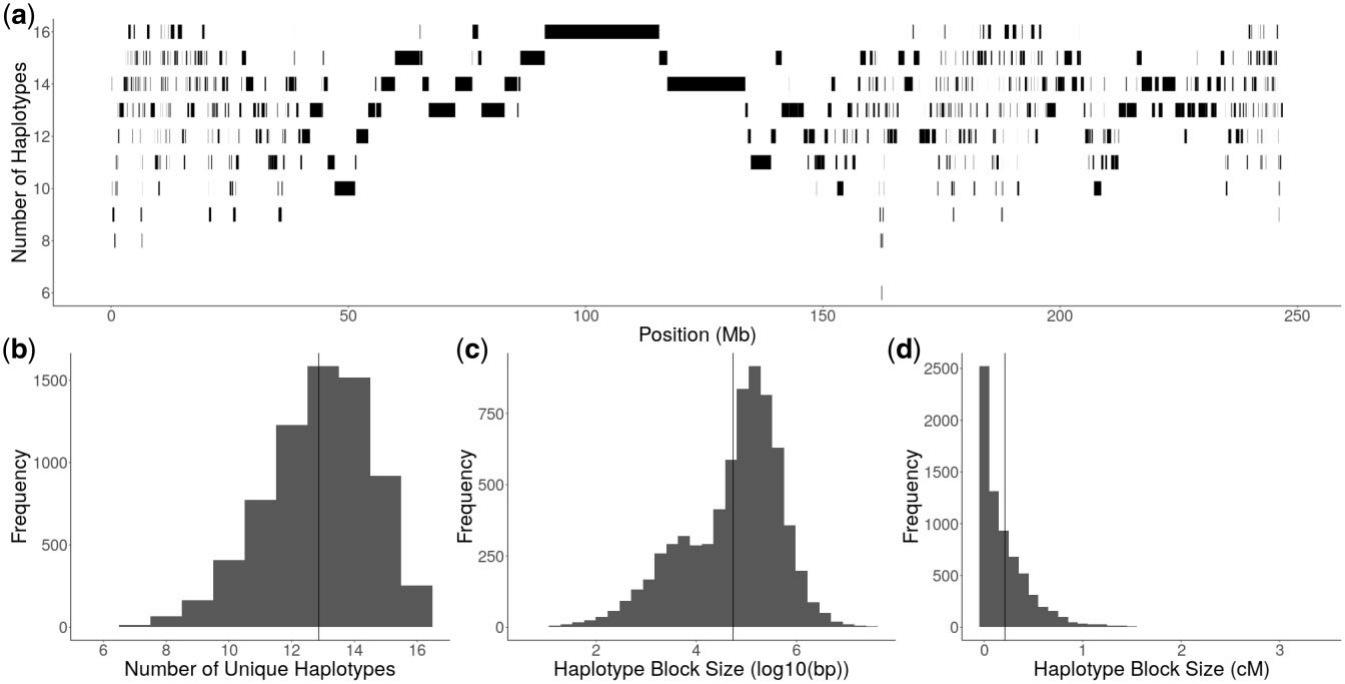
Diversity and size of haplotype blocks. a) The number of unique haplotypes per haplotype block and size of haplotype blocks along chromosome 4 in physical distance. b) Distribution of unique haplotypes per haplotype block across the genome. c) Distribution of haplotype block size in physical distance, represented as log10(bp). The vertical solid line represents the average size of 303.7 kb. d) Distribution of haplotype block size in genetic distance, represented as cM. The vertical solid line represents the average size of 0.2 cM.

Due to the widespread Pairwise IBD between the founders, it appeared that many founders shared ancestral haplotypes. Within individual blocks of ancestry, we collapsed founder alleles that were identical by descent into a single haplotype (see *Materials and methods*). The genome was broken up into a total of 6,929 haplotype blocks. Of those blocks, approximately 16% of them (1,152) contained at least 1 haplotype whose pairwise IBD between parents was incomplete, meaning that there was some genetic variation between founders within those haplotypes that was not captured by the haplotype designation (see *Materials and methods*) (Supplementary Fig. 2). The number of unique haplotypes within haplotype blocks varied across chromosomes, ranging from 6 at the lowest to 16 at the highest (Fig. 3, a and b). The average number of unique haplotypes per haplotype block was 13 (μ=12.85,σ=1.71) (Fig. 3b). There was a wide range of haplotype block sizes, with the average physical size of haplotype blocks being 303.7 kb (σ=1.71Mb) (Fig. 3c). The largest haplotype block was 39.3 Mb long on chromosome 7, which had 16 unique haplotypes. In genetic distance, haplotype block sizes range from 0 to 3.4 cM, with an average of 0.20 cM and a median of 0.11 cM (Fig. 3d).

### Founder representation and linkage disequilibrium

Analysis of the MAGIC population showed that the overall representation of the 16 founders in the MAGIC DH lines was relatively even, with the highest percentage founder, A654, representing 6.7% and the lowest percentage founder, EP1, representing 5.2%, compared to the expectation of 6.25% for each founder (Fig. 2b). However, multiple chromosome regions deviated significantly from the expected equal distribution (Fig. 2a). Across individual regions of each chromosome, 20.4% of the genome significantly deviated from null expectations compared to 100 simulated populations (5% significance threshold $\chi^{2}$ test *P*-value < 1.5e−09) (Supplementary Fig. 14). The fact that the $\chi^{2}$ test performed on 100 simulated MAGIC populations of 344 individuals resulted in far fewer sites with significant allelic imbalance shows that the over- and underrepresentation of certain founder alleles was greater than would be expected by chance. It also shows that over- and underrepresentation of founder alleles in the population was not due to potential inaccuracy of R/qtl2 in assigning founders. These results suggests that a large amount of the over- and underrepresentation of founder alleles in the MAGIC population is biological, rather than a result of model error, and perhaps evidence of selection for or against particular founder alleles.

**Fig. 4.**
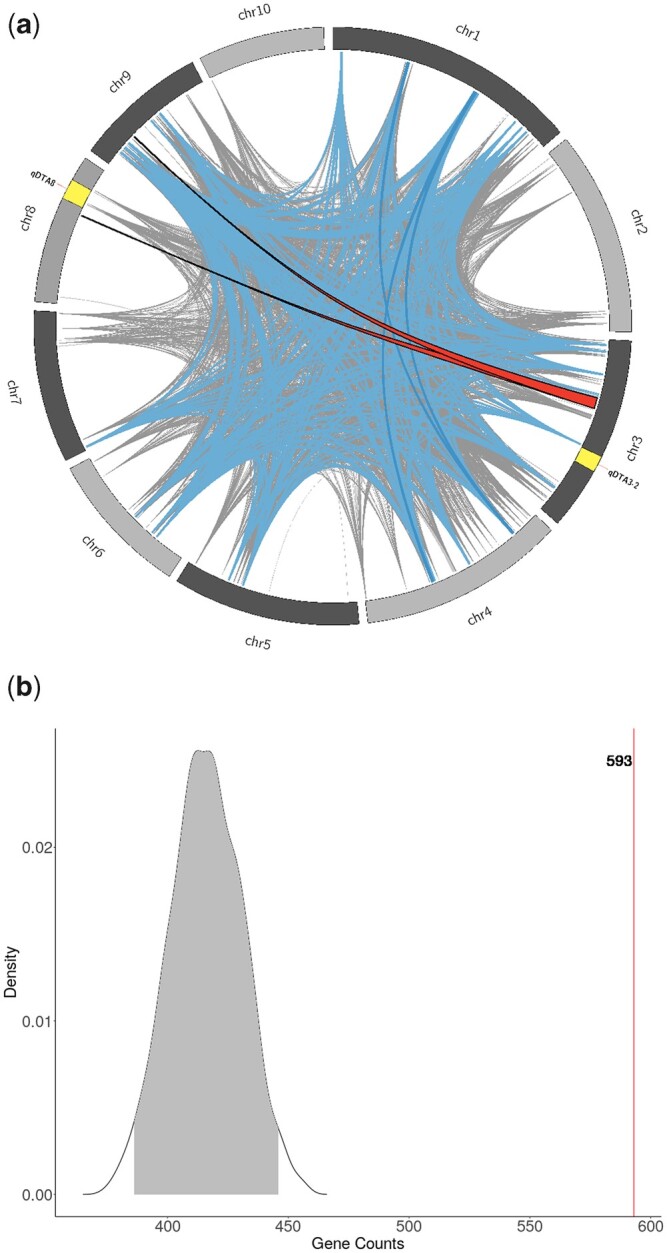
Interchromosomal linkage disequilibrium and flowering time genes in the MAGIC population. a) Ribbons represent regions of *R*^2^ > 0.9 between consecutive SNPs on different chromosomes. The 2 red bands outlined in black are regions >5 Mb on at least 1 of the chromosomes. Blue bands are regions between 1 and 5 Mb one both chromosomes. Gray lines are regions between 100 kb and 1 Mb. The yellow regions represent the support intervals for the flowering time QTL, *qDTA3-2* and *qDTA8*. b) The null distribution of the number of genes overlapping with regions of high interchromosomal LD (*R*^2^ > 0.9) from 887 randomly selected genes from 1,000 permutations. Observed number of flowering time genes overlapping with regions of high interchromosomal LD are shown in red (*n* = 593).

We looked at patterns of linkage disequilibrium in the MAGIC population. The intrachromosomal LD structure showed fast LD decay consistent with many recombination events (Supplementary Fig. 1). Unexpectedly, there was a large amount of high interchromosomal LD (Fig. 4a). Of a total of 9,796,630 SNP pairs with an *R*^2^ greater than or equal to 0.9, 426,178 (4.3%) of those pairs came from different chromosomes. The number of interchromosomal high LD regions was more than would be expected by chance: in 100 simulated populations, there were no SNP pairs with *R*^2^ greater than 0.9 detected between chromosomes. We detected large segments of interchromosomal LD between chromosome 3 and chromosome 8, but these regions did not overlap with the support intervals for *qDTA8* and *qDTA3-2*, corresponding to *vgt1* and *vgt3*, respectively (Fig. 4a).

Because individual DH lines were required to overlap in FT with the tester, MBS847, in order to successfully make F1s for the MAGIC population (Fig. 1), we hypothesized that the patterns observed in founder representation and linkage disequilibrium might be due to selection on FT. In particular, the tester, MBS847, is a generally later flowering line and is *MITE*^–^ at *vgt1* (Chardon et al. 2004), providing the opportunity for selection against early flowering alleles. As one test of this hypothesis, we asked whether genes involved in flowering were enriched in regions with high interchromosomal LD, and found strong enrichment of FT genes in those regions compared to 1,000 randomized permutations (*P*-value < 0.001) (Fig. 4b). We found no significant enrichment of FT genes within regions with significantly uneven founder representation (Supplementary Fig. 15). The finding that regions with high LD between chromosomes contain more FT genes than would be expected by chance suggests that selection for the combination of particular alleles affecting flowering may have occurred.

### QTL mapping and association mapping

We performed association mapping using 3 models of the allelic state of QTL. The first method, *GWAS_SNP_*, used SNP genotypes obtained from the 600K array, assuming that QTL are bi-allelic. The second method, *QTL_F_*, used probabilities of founder identity in chromosome intervals, assuming that a QTL had as many alleles as founders. The third method, *QTL_H_*, used probabilities of haplotype identity in chromosome intervals, assuming that a QTL had as many alleles as ancestral haplotypes. We performed QTL mapping separately in each of the 42 environment: phenotype combinations, plus the across-environment averages of the 7 traits, for a total of 49 separate analyses. The 3 methods varied in their ability to identify QTL. The majority of QTL were identified by all 3 models using a 5% significance threshold (26 or 57%), with 6 QTL found in both *QTL_F_* and *QTL_H_* and 1 QTL found in both *GWAS_SNP_* and *QTL_F_* (Supplementary Table 1 and Supplementary File 4). In addition, each model found unique QTL, with 7, 3, and 3 QTL found in only *GWAS_SNP_*, *QTL_F_*, and *QTL_H_*, respectively (Supplementary Fig. 7 and Supplementary File 5).

Next we merged QTL of the same phenotype from different environments based on overlapping support intervals. There were 20 unique across-environment QTL identified from all methods at a 5% significance threshold, and 10 of these were found in more than 1 environment. We found 12 across-environment QTL using BLUPs and 2 of these were not identified in any individual environment. There were also multiple across-environment QTL that were only found in 1 environment; for example, there were 3 QTL that were only found at a 5% significance threshold in Graneros, Chile, (*qHGM2*, *qDTA7*, and *qTKW7-2*) (Supplementary Table 2).

**Fig. 5.**
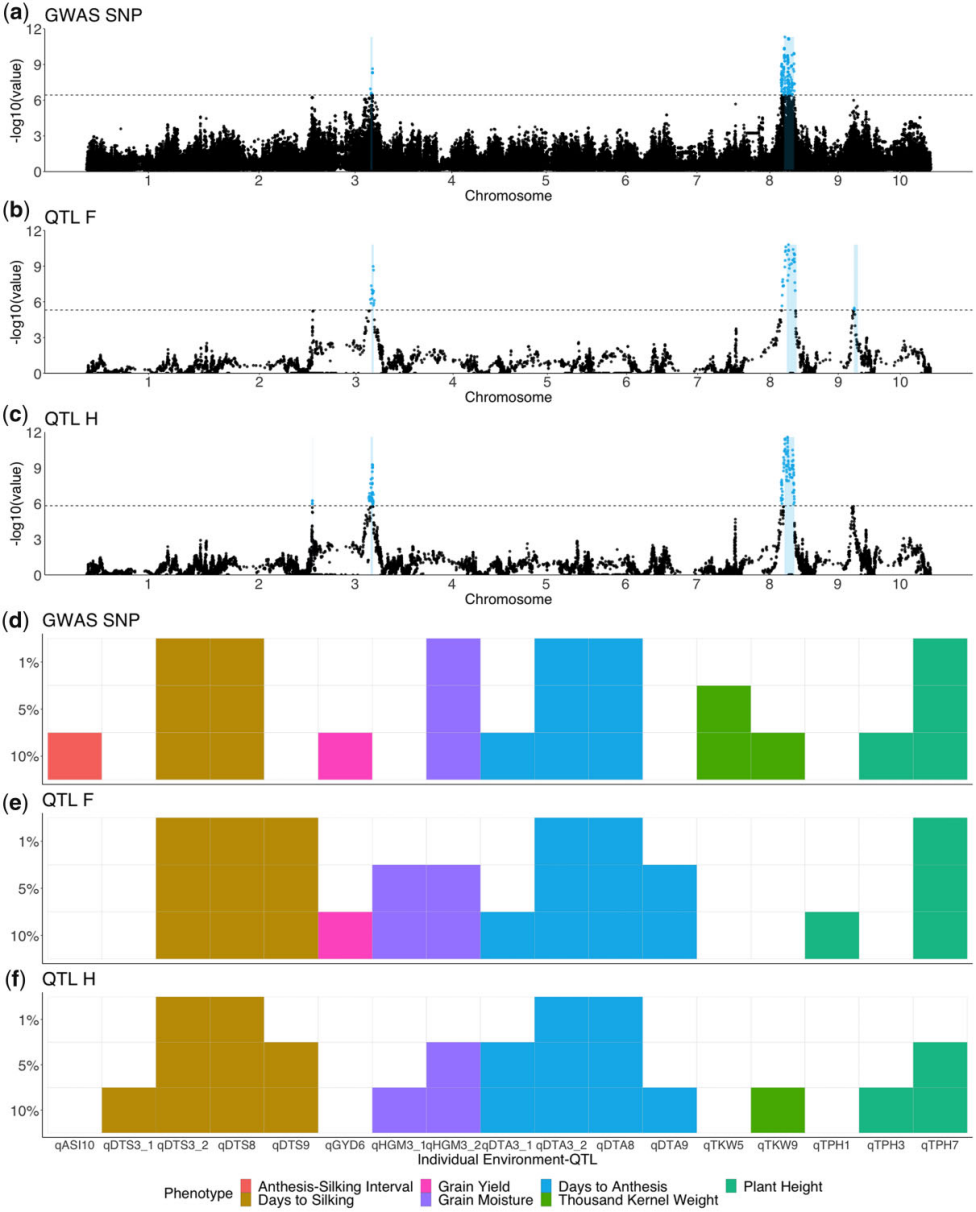
Results of 3 methods of QTL identification. a–c) Manhattan plots for DTA BLUPs. Colored points represent significant QTL above the 5% significance threshold from 1,000 random permutations, located between log10(*P*-value) = 5 and log10(*P*-value) = 6 (dashed line). a) GWAS results using the *GWAS_SNP_* method. b) Results from QTL mapping using the *QTL_F_* method. c) Results from QTL mapping using the *QTL_H_* method. d–f) Identification of individual QTL with varying permutation significance thresholds of 1%, 5%, and 10% across the 3 methods. Different colors indicate the phenotype of the QTL and lighter colors indicate that a QTL was significant at that threshold for a particular method for *GWAS_SNP_* d), *QTL_F_* e), and *QTL_H_* f).

Figure 5, a–c shows the Manhattan plots from the 3 methods for BLUP DTA using a 5% significance threshold. Analysis with individual environments identified fewer QTL, but displayed similar patterns (Supplementary File 2). For DTA, all 3 methods easily identify 2 large QTL, *qDTA3-2* on chromosome 3 and *qDTA8* on chromosome 8. These QTL correspond to 3 previously identified QTL, *vgt3* for *qDTA3-2* and *vgt1* and *vgt2* for *qDTA8*. A second QTL on chromosome 3 for DTA, *qDTA3-1*, was only found at 5% significance with the *QTL_H_* method. A DTA QTL on chromosome 9, *qDTA9* was only found at 5% significance using *QTL_F_*. For the other phenotypes, a harvest grain moisture QTL on chromosome 3, *qHGM3-1* was only found at 5% significance using *QTL_F_* and QTL for DTS with overlapping support intervals to *qDTA9* was found in both *QTL_F_* and *QTL_H_*. The *GWAS_SNP_* method was able to identify 1 QTL at 5% significance on chromosome 5 for thousand-kernel weight, *qTKW-5*, that was not found in either *QTL_F_* or *QTL_H_* (Supplementary Table 1).

We detected a strong overlap between a harvest grain moisture QTL, *qHGM3-2*, with a large FT peak on chromsomes 3 (*qDTA3-2* and *qDTS3-2*). To test if these 3 QTL could be explained by the same causal variants, we looked to see if the estimated effect sizes of the most significant hits from the *GWAS_SNP_* and *QTL_F_* methods were correlated between individuals in the population. It is expected that DTA and DTS will share most of their QTL, as the 2 traits are strongly correlated. As expected, the effect sizes for *qDTS3-2* and *qDTA3-2* were highly correlated using *QTL_F_* (*r* = 0.974) and less so using *GWAS_SNP_* (*r* = 0.492). For *qDTS3-2* and *qHGM3-2*, the effect sizes were moderately correlated, with *r*-values of 0.443 and 0.403 for *QTL_F_* and *GWAS_SNP_*, respectively. The correlations for *qDTA3-2* and *qHGM3-2* were slightly higher (*r* = 0.560) for both *QTL_F_* and *GWAS_SNP_*. Although there is positive correlation between effect sizes at these FT and HGM QTL, there is not enough evidence to definitely say that they share the same causal variant.

Despite differences in the models, the power to identify and refine the location of QTL was similar across the 3 methods. *QTL_F_* was able to identify the most QTL, regardless of changes in the significance threshold (Supplementary Fig. 8). There were 8 environment-QTL (7 unique) for which the QTL that were found in 1 method at the 5% significance threshold became significant in other methods when the 10% threshold was used, indicating that the differences in the ability to detect these QTL between methods was mostly due to differences in power (Fig. 5, d–f and Supplementary File 5). Nonetheless, there were multiple QTL that were identified in only 1 method. There were 8 environment-QTL (all unique) that were significant at the 5% threshold in only 1 method, and did not break significance at the 10% threshold in the other methods. For these QTL, the success of a particular model is most likely due its ability to better fit the allelic variation underlying the QTL; 6 of these 8 QTL were identified by the *GWAS_SNP_* model, suggesting that truly bi-allelic causal variants explain the phenotypic variation at these sites. Similarly, there were 7 environment-QTL (5 unique) that broke significance in all but 1 method, and 6 of these 8 were never identified in *GWAS_SNP_*. This further emphasizes that the success of a bi-allelic model is dependent on the genetic architecture of the QTL, and that applying only this model has the potential to miss true signals.

Support intervals for QTL were determined using a cutoff of a 2log_10_ drop from the *P*-value the most significant site (see *Materials and methods*). The size of *GWAS_SNP_* support intervals was significantly larger than *QTL_H_* support intervals in genetic distance (1.55 cM, SE = 0.56, *t*-ratio = 2.77, *P*-value = 0.021), but the difference in physical distance was not significant (*t*-ratio = 1.97, *P*-value = 0.13) (Supplementary Table 2). There was no significant difference in the size of QTL support intervals between *QTL_F_* and *QTL_H_* in either physical and genetic distance. Although on average, the physical and genetic size of *GWAS_SNP_* support intervals were larger than those of *QTL_F_* support intervals, the difference was not significant, perhaps because of a single outlier QTL in *QTL_F_* with a very large support interval (Supplementary Figs. 9 and 10). When the outlier was dropped from the model, the difference between *GWAS_SNP_* and both *QTL_F_* and *QTL_H_* support intervals were significant in both genetic (*t*-ratio = 2.87, *P*-value = 0.017; *t*-ratio = 3.17, *P*-value = 7.4e−3) and physical distance (*t*-ratio = 2.91, *P*-value = 0.015; *t*-ratio = 2.68, *P*-value = 0.027).

### Variation around *vgt1*

One notable QTL that was identified by all 3 models using BLUPs (Fig. 5) and nearly all individual environments was *qDTA8*, a large QTL on chromosome 8 that was strongly correlated with variation in days to anthesis as well as days to silking. The support interval for this QTL overlapped with 2 previously characterized FT QTL, *vgt1* and *vgt2*. As a well-studied, large effect QTL, *vgt1* provides a useful benchmark for comparison of the 3 allelic models.

**Fig. 6.**
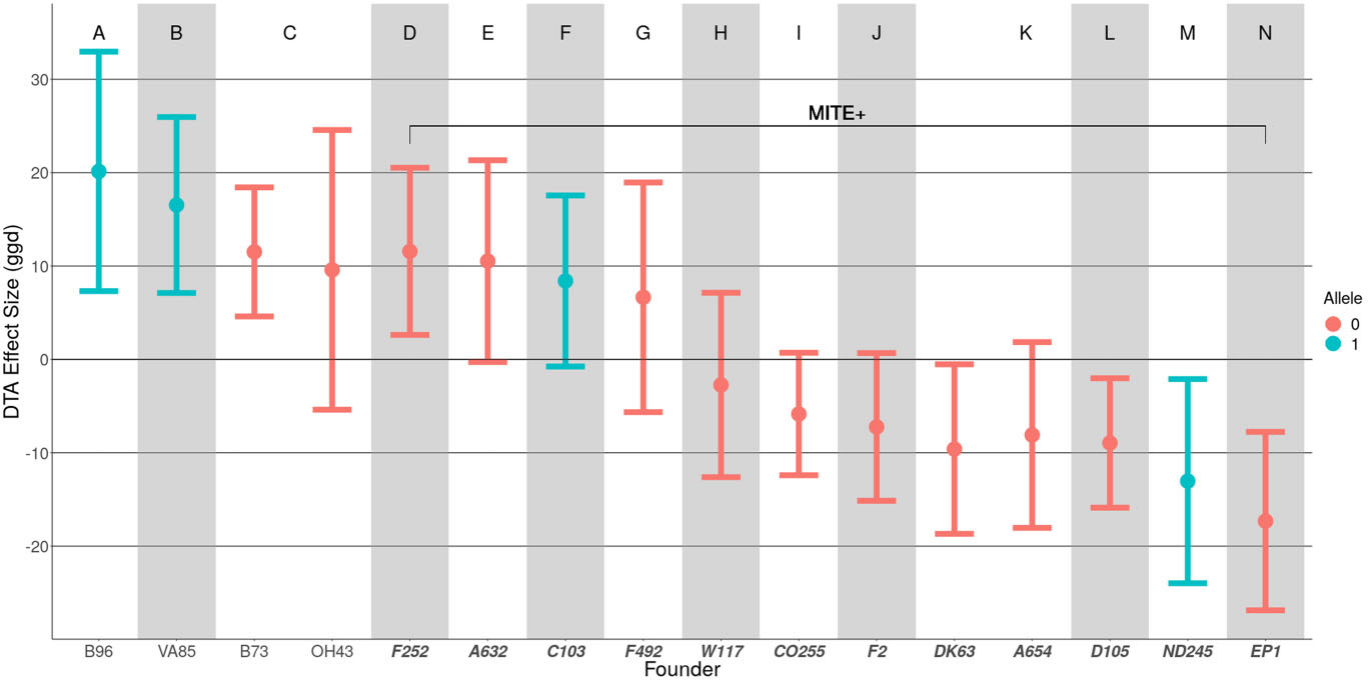
Estimated founder effect sizes for *vgt1*. Estimates of founder effect sizes relative to the population mean for DTA BLUP phenotypes using *QTL_F_*. Letters A–N and groupings of shaded blocks along the *x*-axis indicate founders that have shared haplotypes at the most significant *QTL_F_* SNP. Color indicates reference (0) and alternate (1) alleles at the most significant *QTL_F_* SNP. Positive effect sizes indicate later flowering and negative effect sizes indicate earlier flowering. Founders on the right (D–N) within the bracket are lines that possess the MITE insertion (*MITE*^+^), while founders on the left (A–C) lack the MITE insertion (*MITE*^–^).

At *vgt1*, the 16 founders are segregating (*MITE*^+^/*MITE*^–^) for the putative causal variant, a MITE insertion in a conserved non coding sequence upstream of *ZmRap2.7*, a FT regulatory gene (Salvi et al. 2007; Castelletti et al. 2014). Four founders, B73, OH43, VA85, and B96, lack the MITE insertion, while the other 12 are *MITE*^+^. Looking at the most significant SNP for *qDTA8* from *GWAS_SNP_*, the alternate allele correlated imperfectly with the presence of the MITE in the founders (*r* = 0.65). We expected to see *QTL_F_* effect sizes at this locus that match the allelic state of the founders, with *MITE*^+^ founders having earlier effect sizes and *MITE*^–^ founders having later effect sizes. However, for some founders, the *QTL_F_* effect sizes at *vgt1* deviated from those expectations (Fig. 6). Four *MITE*^+^ founders, A632, F252, C103, and F492, had DTA BLUP effect size estimates later than the population average. While only F252 had a 95% confidence interval not overlapping zero, all had effect sizes significantly later than the other *MITE*^+^ founders (*t*-ratio = 7.67, *P*-value <1e−4). This pattern was also seen in the effect sizes estimated in individual environments (Supplementary File 3). Finally, at the most significant hit from *QTL_H_*, founders are grouped into haplotypes consistent with their allele at the MITE, but there are still far more than 2 distinct haplotypes (14). Analysis of the haplotype structure in the region around *vgt1* in the 16 founders showed clear differences between those that do and do not have the MITE insertion, but did not differentiate *MITE*^+^ late founders from *MITE*^+^ early founders (Supplementary Fig. 11).

One possible explanation for this observation is an epistatic interaction between *vgt1* and other loci in the genome. However, a genome scan for epistasis between *vgt1* and other loci did not yield any significant interactions (Supplementary Fig. 12). *QTL_F_* using only MAGIC lines predicted to have the MITE had 2 significant DTA BLUP QTL in the region of *vgt1* (Supplementary Fig. 13). One of these significant sites is located in close proximity to the causal gene for *vgt2*, *ZCN8*, and may be explained by this linked QTL (Guo et al. 2018). The second significant site is located 15 Mb downstream of *vgt1*, suggesting that some local variation around the region of *vgt1* impacts the effect of the QTL on FT. This site may have an epistatic interaction with *vgt1* that did not pass the stringent genome-wide significance threshold. Alternatively, the relationship between the loci could be entirely additive, but the causal allele may only occur on the *MITE*^+^ background.

## Discussion

We used 3 models of QTL allelic states to identify QTL in the MAGIC population, a bi-allelic model (*GWAS_SNP_*), a founder multiallelic model (*QTL_F_*), and an ancestral haplotype multiallelic model (*QTL_H_*). The *GWAS_SNP_* method should be most powerful at identifying QTL for which the causal variant is bi-allelic and the tagged SNP is in tight LD with the causal variant. However, for multiallelic QTL or QTL for which LD is low between tagged SNPs, this method should have lower power. *QTL_F_*, which assumes that all founders possess distinct alleles, increases the odds of detecting both QTL that are multiallelic and QTL whose causal variant is not in tight LD with any 1 tagged SNP. While the higher number of parameters that must be fit by this model may also reduce power, because the regions tested are much larger than 1 SNP, it also reduces the multiple testing burden. Finally, *QTL_H_* potentially improves on the power of *QTL_F_* to detect QTL that meet the above criteria by reducing the number of parameters that must be estimated. There is, however, the potential for *QTL_H_* to obscure the signal of some QTL if founders are called as having the same ancestral haplotype when they actually differ for a causal variant. Due to the fact that *QTL_H_* and *QTL_F_* only take into account recent recombination events, whereas *GWAS_SNP_* uses historical recombination, we predicted that *GWAS_SNP_* would result in higher resolution of QTL support intervals. Higher resolution QTL are ideal in that they make it easier to narrow down candidate genes and potential causal variants.

The results of using these 3 models of genetic architecture to identify QTL suggest that each has its own advantages and disadvantages in terms of how many and which QTL they can identify. Overall, the *QTL_F_* model performed the best in terms of the number of QTL identified, although there were multiple QTL identified uniquely in all models. In addition, most of the QTL identified by *QTL_F_* at a 5% significance threshold became significant in either one or both of the other methods at a 10% threshold (Fig. 5, d–f). These differences in power may be related to differences in the number of tests performed by each model, or to varying abilities of the models to detect QTL with differing numbers of alleles.

The larger size of *GWAS_SNP_* support intervals was unexpected, as this method is often used to fine map QTL regions identified by linkage mapping. We suspect that this finding is either the result of the somewhat naive method used to determine QTL support intervals, or residual long-distance LD caused by the funnel crossing scheme. Using a 2log_10_ drop from the most significant *P*-value heavily penalizes QTL that just pass significance. In addition, the 100 Mb window used is blind to QTL peaks that contain multiple QTL, although the 2 largest QTL peaks identified, *qDTA3-2* and *qDTA8*, each appeared to only contain one QTL. Overall, our method of defining QTL support intervals, though useful for providing a means of comparison across the 3 methods, is arbitrarily defined. It is unclear how these support intervals compared to a standard 95% confidence interval. As a result, it is difficult to determine a reason for the difference in support interval size between the models.

Previous studies have used variations of these methods to identify QTL, and some have directly compared them. The use of combined linkage and association analyses, sometimes referred to as linkage disequilibrium–linkage association (LDLA), was first proposed by Meuwissen and Goddard (2001), who used predicted IBD probabilities between parents using an evolutionary model and applied them to linkage mapping. LDLA has been used in multiple studies to enhance QTL detection in multiparent populations in maize (Yu et al. 2008; McMullen et al. 2009; Giraud et al. 2017) and other organisms such as pigs (Hérault et al. 2018), rice (Stadlmeier et al. 2019; Zaw et al. 2019), barley (Sannemann et al. 2015), and winter wheat (Stadlmeier et al. 2019). Jansen et al. (2003) used a haplotype-based method for QTL mapping and showed through simulation that this strategy could reduce the number of estimated parameters and, therefore, increase power. Yalcin et al. (2005) compared a bi-allelic model to an ancestral haplotype model in a multiparent mouse population, as well as a “merge analysis” to attempt to identify candidate causal variants underlying QTL. A similar analysis in the outbred NIH-HS rat population used merge analysis and found that many QTL could not be explained by a single causal variant, suggesting a multiallelic basis for these QTL (Baud et al. 2013).

Different means of determining ancestral haplotype blocks from parental sequences have been used, with clusthaplo (Leroux et al. 2014), an extension of the software MCQTL (Jourjon et al. 2005), being a commonly used algorithm in recent studies. Bayesian frameworks have also been implemented in real (Pérez-Enciso 2003) and simulated (Bink et al. 2012) multiparent populations. Giraud et al. (2014) used both a haplotype- and founder-based approach in 2 nested association mapping populations of Northern European flint and dent maize lines created by Bauer et al. (2013) and genotyped with a 50K SNP array (Ganal et al. 2011). Giraud et al. (2014) used clusthaplo to determine haplotype blocks based on IBD between parents and used discrete founder and haplotype values in their models.

Interestingly, the performance of bi-allelic, founder, and ancestral haplotype models differs across studies. Giraud et al. (2014) found that their haplotype model outperformed the founder and SNP models in terms of the number of QTL identified using EU-NAM Flint and Dent maize populations. In contrast, Garin et al. (2020) found that in the EU-NAM Flint population, the bi-allelic model detected a larger number of unique QTL, compared to parental or ancestral haplotype models. Bardol et al. (2013) found that in 2 multiparent dent populations, their bi-allelic model and ancestral haplotype model generally outperformed the parental linkage model, although benefits of these models varied by dataset. The performance of the 3 models seems to depend heavily on the diversity of the parents used to generate the population. For populations with more diverse founders, it would be expected that there would be fewer shared haplotypes between founders, reducing the efficacy of a haplotype model (Giraud et al. 2014). The fact that the *QTL_F_* model outperformed the *QTL_H_* and *GWAS_SNP_* in our population suggests that the MAGIC population contains a relatively more diverse representation of temperate maize than populations used in previous studies. It is also possible that the structure of multiparent populations has an effect on the performance of the 3 models, compared to previous studies which used nested association mapping (Giraud et al. 2014; Garin et al. 2020) and factorial populations (Bardol et al. 2013).

Differences in the estimated effect sizes across models offer suggestions as to the reason for their differences in QTL detection. QTL that were only found in the *GWAS_SNP_* method most likely have a bi-allelic causal variant. It is likely that the increased number of parameters in the *QTL_F_* and *QTL_H_* models reduce statistical power when the true number of functional alleles is low. Similarly, we predict multiallelic QTL were more likely to be identified by the *QTL_F_* or *QTL_H_* models and not the *GWAS_SNP_* method unless the effect size was large. For QTL that were identified in the *QTL_H_* method and not the *QTL_F_* method, there tended to be a lower number of unique ancestral haplotypes, suggesting that *QTL_H_* was more successful in finding these QTL due to improved power when there were fewer functional alleles than founders. For QTL that were not identified by the *QTL_H_* method, particularly for QTL that were successfully identified by *QTL_F_*, there are 2 possible explanations. Because there were more regions tested for *QTL_H_* than for *QTL_F_*, if the true number of ancestral haplotypes at the QTL is large, the *QTL_H_* method may actually have lower power than *QTL_F_* because the number of tests is higher. Alternatively, it may be the result of a failure of *QTL_H_* to accurately represent the true haplotype structure of the QTL region. Our algorithm for defining haplotype blocks relies on a threshold for IBD that is, by nature, somewhat arbitrary. A more lenient cutoff would provide a larger reduction in model parameters by grouping more founders into haplotypes, but runs the risk of washing out the signal of true causal differences between founders. Alternatively, a more stringent threshold would separate the founders into more haplotype groups. This may reduce the efficacy of the *QTL_H_* method because the improvement in statistical power only occurs when the number of haplotype groups is low.

On the whole, most QTL were found by all 3 methods, so there was limited ability to draw reliable conclusions about underlying mechanisms that caused the methods to perform better or worse. Generally, the comparison of QTL detection and effect size estimates suggested that the methods failed and succeeded on a QTL-by-QTL basis. This is to be expected, as each QTL is the result of a distinct set of 2 or more causal alleles with a unique evolutionary history and pattern of linkage disequilibrium within the population.

Whether QTL that appeared in only 1 method are due to false positives or true differences in the methods’ abilities to identify QTL with different genetic architectures cannot be determined, but many of the QTL identified in the MAGIC population have underlying candidate genes or have been found in previous studies, providing support to their biological reality. Multiple FT QTL support intervals overlap or are close to previously identified FT genes and QTL. *qDTA9* is nearby the previously identified maize FT gene *ZmCCT9* (Huang et al. 2018). *qDTA3-2* overlaps with *vgt3*, whose underlying gene was identified as *ZmMADS69* (Liang et al. 2019). *qDTA3-1* is nearby a recently identified FT QTL also associated with phosphatidylcholine levels (Rodríguez-Zapata et al. 2021). The support interval for *qDTA8* overlaps with 2 FT QTL, *vgt1*, which we discuss in length, and another, *vgt2*. The causal gene for *vgt2* is *ZCN8*, which is the maize ortholog of FT in *Arabidopsis* (Lazakis et al. 2011). Variation in the promoter region of *ZCN8* between temperate maize and teosinte suggests that earlier flowering alleles were under selection during the process of maize domestication (Bouchet et al. 2013; Guo et al. 2018). It is interesting to note that there is strong overlap in the support intervals of QTL found on chromosome 3 between FT and harvest grain moisture (Supplementary Table 1), perhaps due to developmental pleiotropy linking FT and the moisture of kernels at harvest, although we cannot say with certainty that these QTL are explained by the same causal variant. Three GxE QTL were detected in this population (Hudson et al. in prep) using the *QTL_F_* model, but none overlapped with the main effect QTL we detected in this study. One of these, QTL appeared to be a false-positive resulting from low representation of one of the founders in the region, indicating the potential for low founder sample size to confound QTL results.

Due to the fact that the MAGIC population is segregating for *vgt1*, it provides an opportunity to further study the mechanism behind the QTL’s affect on FT. One benefit of using founder and haplotype approaches lies in the potential to dissect the effects of individual founders and haplotypes within QTL. This allowed us to look more closely at *vgt1* and observe an interesting pattern of effect sizes that deviated from our expectations based on previous research. Previous research has shown that variation in FT at this site is strongly correlated with a MITE insertion about 70 kb upstream of the FT regulator, *ZmRAP2.7*, an *APETALA*-like transcription factor, with the presence of the MITE associated with an earlier FT (Castelletti et al. 2014). Within maize heterotic groups, Flint maize lines tend to possess the early-flowering allele of *vgt1* (*MITE+*), while dents (such as B73) tend to carry the late-flowering allele (*MITE–*) (Salvi et al. 2007). In addition to being a crucial agronomic trait, FT contributes to local adaptation for annual plants such as maize, ensuring that individuals can reproduce within the growing season of their environments. The frequency of the MITE in maize populations follows a latitudinal gradient, suggesting that the early *MITE*^+^ allele was selected for during the process of maize adaptation to temperate climates (Navarro et al. 2017). It has also been shown that there are differentially methylated regions around *vgt1* between B73, landrace maize, and its wild relative, teosinte (Xu et al. 2020). The hypothesized mechanism of action is that the MITE represses expression of the negative FT regulator, *ZmRAP2.7*, possibly due to changes in methylation around the insertion, resulting in earlier induction of flowering (Castelletti et al. 2014). However, the MITE has not yet been experimentally shown to result in earlier flowering. A recent study using multiple multiparent populations suggested that variation in the effect of *vgt1* in different genetic backgrounds was due to local genetic variation surrounding *vgt1*, rather than epistasis with distant loci (Rio et al. 2020). The observed lack of significant epistasis with *vgt1* in this study (Supplementary Fig. 14), combined with our results of $\mathrm{MIT}E^{+}\;QTL_{F}$ (Supplementary Fig. 12), appear consistent with this idea. This finding suggests 2 possibilities: either (1) that the causal variant underlying *vgt1* is some as-yet unidentified variant that is in tight, but imperfect linkage disequilibrium with the MITE insertion, or (2) that the MITE insertion directly impacts FT, and that another variant nearby has a modifying effect on the MITE. This opens up new areas of inquiry for future studies. Mathew et al. (2018) used a barley population (Sannemann et al. 2015) to detect epistatic interactions in FT using a Bayesian framework, and a similar method could perhaps be applied here.

The population displayed relatively high levels of interchromosomal LD (Fig. 4a), which deviated significantly from those obtained from simulations. A potential consequence of interchromosomal LD is the chance for confounding of association analyses, namely resulting in the detection of “ghost” QTL. For the discussed QTL that were somewhat near high interchromosomal LD regions, *qDTA8* and *qDTA3-2*, their effects on FT were independent (Fig. 4a). Nonetheless, the chance for false-positives and inaccurate support intervals due to LD structure is still worth noting. Interchromosomal LD has been detected in multiple populations of domesticated organisms, where breeding has resulted in the preservation of certain combinations of favorable alleles between chromosomes (Malysheva-Otto et al. 2006; Robbins et al. 2011). Strong selection and positive or negative epistasis in natural populations have also been shown to create a pattern of interchromosomal LD (Petkov et al. 2005; Kulminski 2011; Hench et al. 2019; Gupta et al. 2021). Both of these observations suggest that forces other than those of random segregation have operated on the MAGIC population, further supported by the fact that genes affecting FT were strongly enriched in regions of high interchromosomal LD (Fig. 4b). These observations raise the possibility that the complex crossing schemes of MAGIC populations have the potential to introduce the influence of selection, which may restrict the very genetic variation that we are attempting to study.

### Conclusion

The MAGIC population presented here provides a useful resource for investigating quantitative trait variation in temperate maize. As a multiparent population, it has the advantages of increased genetic diversity and higher power to detect QTL with lower allele frequencies. Simulations of the MAGIC population provide an opportunity to validate assignment of founder identities, as well as generate null expectations for various aspects of the population. Overall, we find that a founder multiallelic model identifies the most QTL, although all 3 models of allelic state are effective at identifying QTL. The benefits of increasing statistical power by reducing model parameters in bi-allelic and ancestral haplotype models seem to be tempered by the true allelic complexity of the multiparent population being studied. We conclude that if the goal of a study is to find as many QTL as possible, then it would be most useful to consider the genetic diversity contained within the population when choosing a model in order to maximize QTL identification.

## Data availability

Genotypic data will be made available through FigShare. Phenotypic and environmental data will be made available at FigShare associated with our companion paper (Hudson et al. in prep) (tracking number G3-2021-402688) (https://figshare.com/s/5ee8337defdef63b04ce). Supplementary files are available at FigShare. Supplementary File 1 contains a detailed description of the 16 founders and the crossing scheme used to develop the MAGIC population. Supplementary File 2 shows Manhattan plots similar to Fig. 5, a–c for individual phenotypes and environments. Supplementary File 3 shows founder effect size plots for *vgt1* similar to Fig. 6 for individual environments. Supplementary File 4 contains tables of all QTL identified in the study, their locations, and their effect sizes estimated from the 3 models. Supplementary File 5 shows colored heatmaps similar to Fig. 5, d–f for individual phenotypes and environments. Code used to run analyses and to generate simulated data can be found at https://github.com/sarahodell
